# *Dll1* Haploinsufficiency in Adult Mice Leads to a Complex Phenotype Affecting Metabolic and Immunological Processes

**DOI:** 10.1371/journal.pone.0006054

**Published:** 2009-06-29

**Authors:** Isabel Rubio-Aliaga, Gerhard K. H. Przemeck, Helmut Fuchs, Valérie Gailus-Durner, Thure Adler, Wolfgang Hans, Marion Horsch, Birgit Rathkolb, Jan Rozman, Anja Schrewe, Sibylle Wagner, Sabine M. Hoelter, Lore Becker, Thomas Klopstock, Wolfgang Wurst, Eckhard Wolf, Martin Klingenspor, Boris T. Ivandic, Dirk H. Busch, Johannes Beckers, Martin Hrabé de Angelis

**Affiliations:** 1 Institute of Experimental Genetics, Helmholtz Zentrum Muenchen, German Research Center for Environmental Health, Neuherberg, Germany; 2 Institute for Medical Microbiology, Immunology and Hygiene, Technische Universitaet Muenchen, Munich, Germany; 3 Chair for Molecular Animal Breeding and Biotechnology/LAFUGA, Gene Center, Ludwig-Maximilians-Universitaet Muenchen, Munich, Germany; 4 Molecular Nutritional Medicine, Technische Universitaet Muenchen, Else Kroener-Fresenius Center, Freising-Weihenstephan, Germany; 5 Department of Medicine III, Division of Cardiology, University of Heidelberg, Heidelberg, Germany; 6 Institute of Developmental Genetics, Helmholtz Zentrum Muenchen, German Research Center for Environmental Health, Neuherberg, Germany; 7 Friedrich-Baur-Institute, Department of Neurology, Ludwig-Maximilians-Universitaet, Munich, Germany; 8 Lehrstuhl fuer Experimentelle Genetik, Technische Universitaet Muenchen, Freising-Weihenstephan, Germany; 9 Lehrstuhl fuer Entwicklungsgenetik, Technische Universitaet Muenchen, Freising-Weihenstephan, Germany; New York University School of Medicine, United States of America

## Abstract

**Background:**

The Notch signaling pathway is an evolutionary conserved signal transduction pathway involved in embryonic patterning and regulation of cell fates during development and self-renewal. Recent studies have demonstrated that this pathway is integral to a complex system of interactions, involving as well other signal transduction pathways, and implicated in distinct human diseases. Delta-like 1 (Dll1) is one of the known ligands of the Notch receptors. The role of the Notch ligands is less well understood. Loss-of-function of *Dll1* leads to embryonic lethality, but reduction of Delta-like 1 protein levels has not been studied in adult stage.

**Methodology/Principal Findings:**

Here we present the haploinsufficient phenotype of *Dll1* and a missense mutant *Dll1* allele (*Dll1^C413Y^*). Haploinsufficiency leads to a complex phenotype with several biological processes altered. These alterations reveal the importance of *Dll1* mainly in metabolism, energy balance and in immunology. The animals are smaller, lighter, with altered fat to lean ratio and have increased blood pressure and a slight bradycardia. The animals have reduced cholesterol and triglyceride levels in blood. At the immunological level a subtle phenotype is observed due to the effect and fine-tuning of the signaling network at the different levels of differentiation, proliferation and function of lymphocytes. Moreover, the importance of the proteolytic regulation of the Notch signaling network emphasized.

**Conclusions/Significance:**

In conclusion, slight alterations in one *player* of Notch signaling alter the entire organism, emphasizing the fine-tuning character of this pathway in a high number of processes.

## Introduction

The Notch signaling pathway is an intercellular signaling mechanism that is highly conserved during evolution of vertebrates. It is involved in the determination of cell fates in different cell types during embryonic development. First described in the fly, *Notch* orthologs have been identified successively in many species including birds, rodents and man. In mammals four distinct *Notch* genes *(Notch1*–*Notch4*) have been identified. The Notch receptors are anchored to the membrane via a single transmembrane domain. Two families of transmembrane ligands interact with the Notch receptors, the Delta and the Jagged/Serrate proteins. In mammals three Delta and two Jagged/Serrate class proteins have been described. The canonical Notch signaling pathway involves proteolytic processing events that cleave the Notch receptors in response to ligand activation, leading to the activation of target genes (frequently basic helix-loop-helix transcriptional factors) (for review see [1]–[3]).

Loss- or gain-of-function of the Notch receptors or their ligands have diverse and often severe clinical, physiological and biological consequences in humans (for review see [4], [5]). T-cell acute lymphoblastic leukemia, aortic valve disease, and familial forms of cardiomyopathy, have been associated with mutations in *Notch1*. Alagille syndrome, a developmental disease, is associated with mutations in *Jagged1* and in less extent with mutations in *Notch2*. Mutations in *Notch3* lead to a neurological disease, a cerebral arteriopathy autosomal dominant disease with subcortical infarcts and leukoencephalopathy abbreviated as CADASIL. Mutations in *Delta3* have been associated with spondylocostal dysostosis which leads to severe congenital malformations of the vertebral column. Notch signaling and associated disease involve not only these inherited disorders. Recent publications revealed the involvement of Notch signaling in tumor associated angiogenesis (for review see [6], [7]). Experiments in animal models show that the Notch signaling pathway also plays an important role in tissue regeneration after injury of adult organs, such as heart, liver, brain, kidney and pancreas [8]–[12]. Therefore, Notch signaling has many diverse roles in physiology and pathology.

The fine-tuning of this complex system of regulatory interactions with pleiotropic functions is essential to avoid disease states, but not yet fully understood. One efficient approach to identify components of a molecular network is to perform genetic modifier- or sensitized-screens [13]. Recently, we reported the identification of phenotypic modifiers in a sensitized screen using *Dll1* heterozygous (*Dll1^tm1Gos/+^*) animals [14]. A complementary approach to better understand pleiotropic Notch signaling functions is to investigate the haploinsufficient phenotype of Notch pathway members, since most homozygous Notch mutants and mutant alleles of interacting proteins or ligands are embryonic lethal [15]–[19]. Here, we have comprehensively analyzed the phenotype of *Dll1^tm1Gos^* heterozygous animals and of a missense mutant *Dll1* allele (*Dll1^C413Y^*) in 14 phenotyping platforms covering more than 350 parameters at the German Mouse Clinic (GMC) [20]–[22].

Haploinsufficiency was recently investigated for the Notch ligand Jagged1 in two inbred mouse strains with distinct phenotypes [23], so the genetic background of the inbred mouse strain may affect the observable mutant phenotype. We present here the haploinsufficient phenotype of *Dll1^tm1Gos/+^* mice on the C3HeB/FeJ genetic background and compare the results to previously recorded data on the 129X1/SvJ genetic background.

## Results

### Genetic background of Dll1^tm1Gos/+^ mice

We outcrossed the mice carrying the *Dll1^tm1Gos^* allele [15] from a 129X1/SvJ (129X1) genetic background for 11 generations to the C3HeB/FeJ (C3H) genetic background. The 129X1 genetic background was recently identified as being non-isogenic [14]. In the *C3.Dll1^tm1Gos/+^* animals only one of 137 genome-wide SNPs showed heterozygosity (data not shown). This SNP (rs13482869) is at a distance of approximately 5 Mb to the *Dll1* gene in which the *lacZ* reporter gene was inserted to disrupt the *Dll1* coding sequence [15].

### Body weight, size and body composition

**Figure 1:**
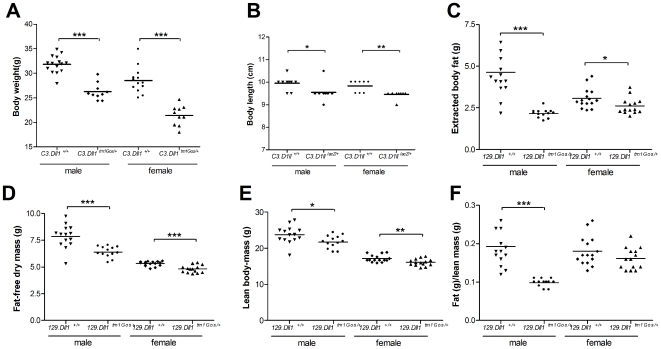
Body weight, size and composition in heterozygous *Dll1^tm1Gos/+^* animals compared to wild-type littermates. A–B) Body weight and body length were determined at 15 and 16 weeks of age, respectively, in two different cohorts. Depicted are the scatter dot plot representations and the mean for each group: heterozygous *C3.Dll1^tm1Gos/+^* males (weight, n = 13; length, n = 9) and their wild-type littermates (*C3.Dll1^+/+^*, weight, n = 15; length, n = 10) and heterozygous *C3.Dll1^tm1Gos/+^* females (n = 10) and their wild-type littermates (*C3.Dll1^+/+^*, weight, n = 10; length, n = 9). C–F) Selected metabolic parameters were recorded using the Soxhlet lipid extraction with petrol ether as solvent in the tertiary screen at the GMC at 30–32 weeks of age. Depicted are the scatter dot plot representations for and the mean for each group: heterozygous *129.Dll1^tm1Gos/+^* males (n = 13) and their wild-type littermates (*C3.Dll1^+/+^*, n = 14); heterozygous *C3.Dll1^tm1Gos/+^* females (n = 14) and their wild-type littermates (*129.Dll1^+/+^*, n = 15). *P*-value calculated performing unpaired t-test when variances were not significantly different. If significantly different then the Mann-Whitney test was performed. *P*-value: * *P*<0.05, ** *P*<0.01, *** *P*<0.001.

*C3.Dll1^tm1Gos/+^* animals are significantly lighter than wild-type littermates (Figure 1A). This difference in body weight is more pronounced in C3H than in 129X1 and mixed (129X1/SvJ.C57BL6/J) genetic backgrounds [14]. Heterozygous C3H females showed on average an 18% to 20% reduction and heterozygous C3H males on average an 11% to 15% reduction in their body weight at 15 weeks after birth (depending on the analyzed cohort, see Figure 1A and Table S1). The average body weights of *C3.Dll1^tm1Gos/+^* mice were also reduced at 6, 8, 10, and 12 weeks of age (Table S1).

Male and female *Dll1^tm1Gos/+^* animals were significantly shorter than their wild-type littermates (Figure 1B). To investigate body composition a cohort of mutant and wild-type 129X1 mice entered the metabolic screen at 30 to 32 weeks of age. No significant differences in the water contents of wild-type and *Dll1^tm1Gos/+^* animals could be detected (data not shown). Using the Soxhlet lipid extraction with petrol ether as solvent the body fat, fat-free dry mass, and lean-body mass of the mice were measured. *129.Dll1^tm1Gos/+^* animals showed a significantly reduced average body fat content (Figure 1C), reduced average fat-free dry mass (Figure 1D) and a reduced average lean-body mass (Figure 1E) when compared to wild-type littermates. Importantly, the average fat to lean mass ratio was significantly reduced in male and slightly reduced in female heterozygous mutants when compared to wild-type animals (Figure 1F). Similarly, *C3.Dll1^tm1Gos/+^ animals* showed reduced body fat and lean mass based upon DEXA densitometry (see Table S1).

In summary, *Dll1^tm1Gos/+^* animals are shorter; show a reduced body weight and an altered body composition, independently from the two genetic backgrounds.

### Energy metabolism

**Figure 2:**
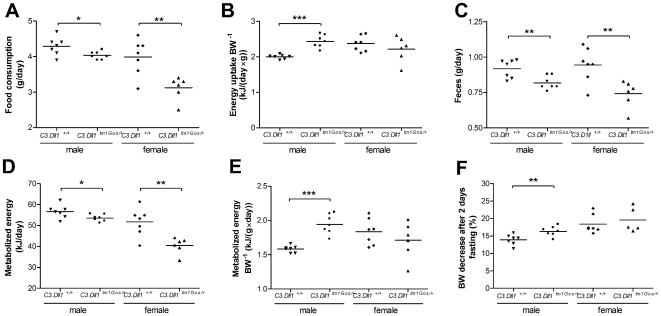
Metabolic parameters collected from heterozygous *C3.Dll1^tm1Gos/+^* animals and their wild-type littermates at the GMC. A–E) Values were recorded during 2 weeks *ad libitum* feeding. F) Body weight decrease (%) after two days fasting. Depicted are the scatter dot plot representations and the mean for each group: heterozygous *C3.Dll1^tm1Gos/+^* males (n = 7) and their wild-type littermates (*C3.Dll1^+/+^*, n = 7); heterozygous *C3.Dll1^tm1Gos/+^* females (n = 6) and their wild-type littermates (*C3.Dll1^+/+^*, n = 7). *P*-value calculated performing unpaired t-test when variances were not significantly different. If significantly different then the Mann-Whitney test was performed. *P*-value: * *P*<0.05, ** *P*<0.01, *** *P*<0.001.

*C3.Dll1^tm1Gos/+^* animals on average consumed significantly less food ad libitum and had a reduced energy uptake as compared to wild-type littermates (Figure 2A). However, when energy uptake was normalized to body weight only the male *Dll1^tm1Gos/+^* mice showed a significant increase of energy uptake in comparison to wild-type males. No such difference in body weight normalized energy uptake was observed between wild-type and *C3.Dll1^tm1Gos/+^* females (Figure 2B).

As a consequence of decreased food intake, both male and female *C3.Dll1^tm1Gos/+^* animals showed significantly reduced feces excretion per day compared to wild-type littermates (Figure 2C). The metabolized energy of individual mice was measured by subtracting the energy content of collected feces and urine from the energy uptake. Both female and male *C3.Dll1^tm1Gos/+^* mice showed significantly reduced metabolized energy (Figure 2D). When normalized to the individual body weights, significantly increased metabolized energy was detected only for the group of *C3.Dll1^tm1Gos/+^* males (Figure 2E). In contrast, in *129.Dll1^tm1Gos/+^* mice only females showed increased energy uptake and metabolized energy compared to wild-type female littermates. The tendency towards this difference in both parameters was also evident for male *129.Dll1^tm1Gos/+^* mice (see Table S2).

To further analyze the differences in metabolized energy, mice of both genetic strains were fasted for two days. Heterozygous animals of both sexes and genetic backgrounds showed a greater loss of body weight than the corresponding controls. This difference was statistically significant for male *C3.Dll1^tm1Gos/+^* and female *129.Dll1^tm1Gos/+^* mice (Figure 2F and Table S2).

In conclusion, our data suggest that *Dll1* heterozygosity causes increased energy requirements.

### The cardiovascular mutant phenotype

**Figure 3:**
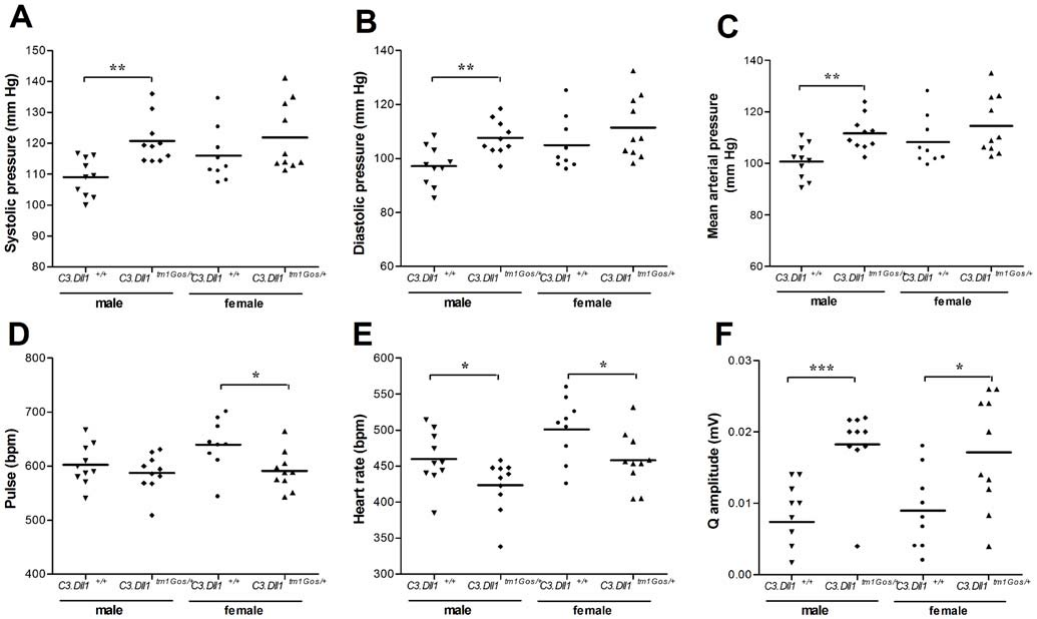
Selected blood pressure and electrocardiogram parameters in heterozygous *C3.Dll1^tm1Gos/+^* animals and their wild-type littermates. A–F) Depicted are the scatter dot plot representations and the mean for each group: heterozygous *C3.Dll1^tm1Gos/+^* males (n = 10) and their wild-type littermates (*C3.Dll1^+/+^*, n = 10); heterozygous *C3.Dll1^tm1Gos/+^* females (n = 10) and their wild-type littermates (*C3.Dll1^+/+^*, n = 9). *P*-value calculated performing unpaired t-test as variances were not significantly different. *P*-value: * *P*<0.05, ** *P*<0.01, *** *P*<0.001.

At the age of 14 weeks *C3.Dll1^tm1Gos/+^* and wild-type littermates were analyzed for cardiovascular parameters. The systolic, diastolic and mean arterial pressures were significantly elevated in *C3.Dll1^tm1Gos/+^* male mice (Figure 3A–C). The resting heart rate was determined during blood pressure measurements (“pulse” in beats per minute, bpm) and in anaesthetized mice during electrocardiography (“heart rate” in bpm). In *C3.Dll1^tm1Gos/+^* female animals the average pulse was significantly, in male mice slightly decreased as compared to wild-type littermates (Figure 3D). The heart rate was significantly decreased in both male and female *C3.Dll1^tm1Gos/+^* animals (Figure 3E), indicating a slight bradycardia in heterozygous mutant animals. Moreover, the Q amplitude was significantly increased in the electrocardiogram traces of heterozygotes (Figure 3F).

### Alteration of clinical chemical blood parameters

**Figure 4:**
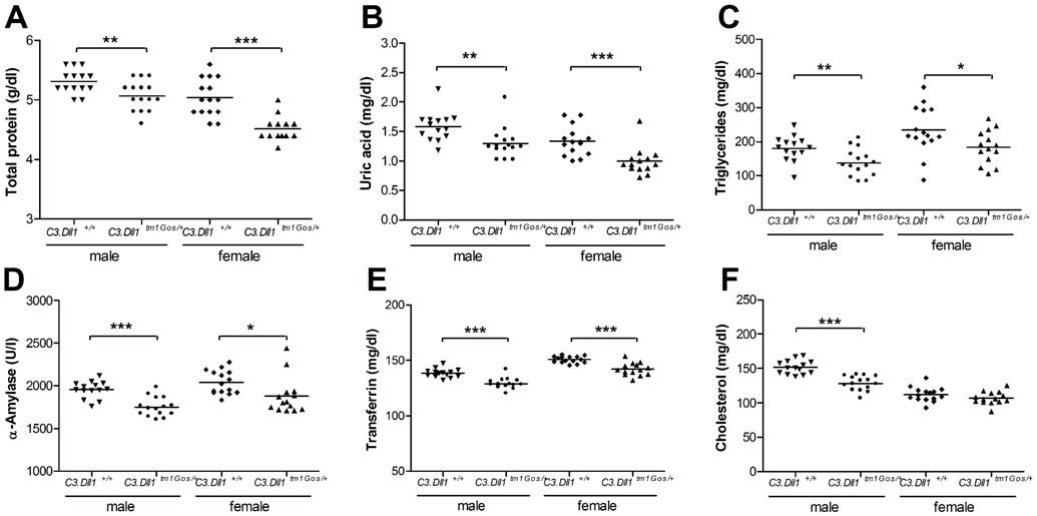
Selected clinical chemistry parameters in heterozygous *C3.Dll1^tm1Gos^* animals and their wild-type littermates. A–F) Depicted are the scatter dot plot representations and the mean for each group: heterozygous *C3.Dll1^tm1Gos/+^* males (n = 15) and their wild-type littermates (*C3.Dll1^+/+^*, n = 14) and heterozygous *C3.Dll1^tm1Gos/+^* females (n = 14) and their wild-type littermates (*C3.Dll1^+/+^*, n = 15). *P*-value calculated performing unpaired t-test when variances were not significantly different. If significantly different then the Mann-Whitney test was performed. *P*-value: * *P*<0.05, ** *P*<0.01, *** *P*<0.001.

At 12 weeks of age blood samples were taken from fed mice for the analysis of 20 clinical chemical parameters (see Table S3). The concentrations of total protein, uric acid, triglycerides and transferrin, as well as α-amylase activity were significantly decreased in male and female *C3.Dll1^tm1Gos/+^* animals (Figure 4A–E). In addition, several other blood parameters were significantly altered in heterozygous mutant mice of only one gender (see Table S3). For example, the average concentration of cholesterol in the blood of *C3.Dll1^tm1Gos/+^* mice was significantly reduced only in male animals (Figure 4F). Taken together, our data demonstrate that heterozygosity for a *Dll1* loss-of-function allele resulted in altered blood concentrations of key metabolites of the protein, carbohydrate and fat metabolism.

### Immunology screen

The same blood samples as above were analyzed by flow cytometry and indirect ELISA. The population of CD19 positive cells (B-cell population) was significantly increased in female but not male *Dll1^tm1Gos/+^* animals of both genetic backgrounds compared to corresponding wild-type littermates (Table 1 and data not shown). Moreover, the proportion of B1 cells within the B cluster was significantly decreased in female *C3.Dll1^tm1Gos/+^* mice. A reduction of the CD4^+^ T-cell population in *C3.Dll1^tm1Gos/+^* mice was significant only in females (Table 1). A similar reduction in CD4^+^ cells was observed in the *129.Dll1^tm1Gos/+^* genetic background, although the difference between wild-type mice and heterozygous mutants was not statistically significant (data not shown). The population of CD8α,β T-cells showed sex-dependent differences. Compared to the results for the corresponding wild-type littermates this cell population was significantly increased in male and significantly decreased in female heterozygous mutant animals (Table 1). IgG_1_, IgG_2b_, IgG_3_ and IgA parameters were significantly decreased in female *C3.Dll1^tm1Gos/+^* animals. In conclusion, our data suggest that haploinsufficiency of *Dll1* causes alterations in immunological parameters, in particular in the number of B-lymphocytes.

**Table 1:** Immunological parameters in heterozygous *C3.Dll1^tm1Gos/+^* animals.

Sex	Parameter	*C3.Dll1^+/+^*	*C3.Dll1^tm1Gos/+^*	P-value	Regulation
**Male**	**CD19^+^ (%)**	13.00±1.63	12.98±2.60	0.98	↔
	**Cd19^+^/CD5^−^ (%)**	86.33±2.16	88.03±1.22	0.044*	↑
	**Cd19^+^/CD5^+^ (%)**	13.69±2.17	11.98±1.21	0.043*	↓
	**CD8a^+^ (%)**	11.67±0.66	10.78±1.27	0.066	↔
	**CD4^+^ (%)**	27.37±3.36	25.29±2.34	0.13	↔
	**CD8a/b^+^ (%)**	9.36±0.92	10.67±1.49	0.036*	↑
	**Gr-1^+^ (%)**	8.73±2.17	8.96±1.97	0.81	↔
	**γ/δ TCR^+^ (%)**	0.40±0.20	0.48±0.21	0.44	↔
	**IgG_1_ (µg/ml)**	60.64±30.94	82.45±32.25	0.085	↔
	**IgG_2a_ (µg/ml)**	219.08±151.26	217.37±147.51	0.98	↔
	**IgG_2b_ (µg/ml)**	78.59±20.35	83.78±33.45	0.61	↔
	**IgG_3_ (µg/ml)**	195.84±88.51	180.41±68.46	0.60	↔
	**IgA (µg/ml)**	320.31±190.92	333.29±222.32	0.87	↔
	**Anti-DNA (%)**	0.40±0.03	0.39±0.04	0.56	↔
	**Rheumatoid. factor (%)**	0.17±0.02	0.16±0.02	0.51	↔
**Female**	**CD19^+^ (%)**	13.70±3.57	20.28±3.32	0.00065***	↑
	**Cd19^+^/CD5^−^ (%)**	77.84±7.89	82.13±2.32	0.15	↔
	**Cd19^+^/CD5^+^ (%)**	22.16±7.89	17.87±2.32	0.15	↔
	**CD8a^+^ (%)**	10.26±1.14	9.64±1.71	0.37	↔
	**CD4^+^ (%)**	31.53±4.27	22.60±3.49	0.00011***	↓
	**CD8a/b^+^ (%)**	10.22±1.12	8.53±1.09	0.0040**	↓
	**Gr-1^+^ (%)**	8.60±1.96	7.86±2.17	0.43	↔
	**γ/δ TCR^+^ (%)**	0.05±0.02	0.05±0.02	0.60	↔
	**IgG_1_ (µg/ml)**	136.10±53.21	72.61±36.40	0.00092***	↓
	**IgG_2a_ (µg/ml)**	331.72±222.39	224.28±138.18	0.16	↔
	**IgG_2b_ (µg/ml)**	152.65±69.31	94.36±58.59	0.022*	↓
	**IgG_3_ (µg/ml)**	302.33±134.89	160.71±80.02	0.0021***	↓
	**IgA (µg/ml)**	377.32±132.22	218.62±164.50	0.014*	↓
	**Anti-DNA (%)**	0.51±0.08	0.44±0.05	0.0099**	↓
	**Rheumatoid. factor (%)**	0.22±0.07	0.18±0.01	0.07	↔

Values displayed as mean±SD. Data obtained by flow cytometry and indirect ELISA. *P-value* calculated performing unpaired t-test with samples with equal variances, if not Mann-Whitney test performed: *<0.05, **<0.01, ***<0.001.

### Dysmorphology screen

At the age of 9 weeks wild-type and *C3.Dll1^tm1Gos/+^* mice were analyzed for 26 dysmorphology parameters (see Materials and Methods). One wild-type animal out of 58 showed a variant phenotype (kinked tail phenotype), whereas their heterozygous *C3.Dll1^tm1Gos/+^* littermates showed seven variant phenotypes (3 small-body size and 4 kinked tail phenotypes, see Table 2). The Fisher's exact test revealed no significant differences between heterozygous *C3.Dll1^tm1Gos/+^* and wild-type animals in the appearance of a variant morphological phenotype. The reduced body weight and body length of *Dll1^tm1Gos/+^* mice were described above.

**Table 2:** Skeletal phenotyping of heterozygous *Dll1^tm1Gos/+^* animals.

Phenotyping screen	Genotype	No variation	Variation	Two-sided P-value
Dysmorphology	*C3.Dll1^+/+^*	58	1*	0.061
	*C3.Dll1^tm1Gos/+^*	52	7	
Quantification lumbar vertebrae	*C3.Dll1^+/+^*	19	0	0.678
	*C3.Dll1^tm1Gos/+^*	19	1	
	*129.Dll1^+/+^*	17	2	
	*129.Dll1^tm1Gos/+^*	16	3	

Displayed are the numbers of animals showing the observed phenotypes.

* One animal was removed of the analysis, as its variant phenotype, an abnormal eye, could be rather due to injury than a genetic component.

The two-sided P-value was calculated using the Fisher's Exact Test or the Chi-square for the genome-wide vs modifier screen, due to the higher number of cases compared.

P-value: *<0.05, **<0.01, ***<0.001.

At 16 to 17 weeks of age 9 to 10 animals of each group (separated by genotype and gender) were investigated with X-ray autoradiography. One heterozygous *C3.Dll1^tm1Gos/+^* animal showed a reduced number of lumbar vertebrae (Table 2). No differences were observed in other parts of the axial skeleton. In the 129X1 background, a different number of lumbar vertebrae was found in heterozygous and wild-type animals. The Fisher's Exact test revealed no significant differences due to the genotype and appearance of an abnormal number of lumbar vertebrae.

**Figure 5:**
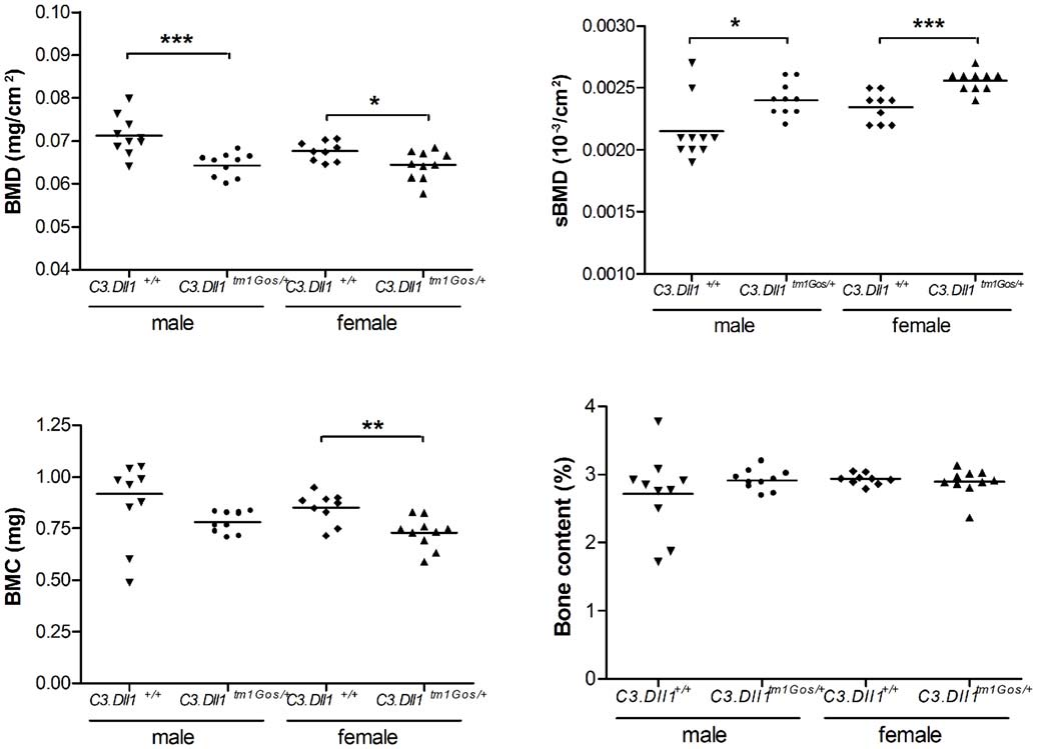
Selected bone parameters obtained with a Dual energy X-ray absorptiometer from heterozygous *C3.Dll1^tm1Gos/+^*animals. A–D) Depicted are the scatter dot plot representations and the mean for each group: heterozygous *C3.Dll1^tm1Gos/+^* males (n = 10) and their wild-type littermates (*C3.Dll1^+/+^*, n = 10); heterozygous *C3.Dll1^tm1Gos/+^* females (n = 9) and their wild-type littermates (*C3.Dll1^+/+^*, n = 10). *P*-values calculated performing unpaired t-test when variances were not significantly different. If significantly different then the Mann-Whitney test was performed. *P*-value: * *P*<0.05, ** *P*<0.01, *** *P*<0.001.

Further bone parameters were measured using DEXA densitometry. Whole body bone mineral density (BMD) was significantly reduced in the *C3.Dll1^tm1Gos/+^* animals compared to their wild-type littermates (Figure 5A). Nevertheless, when related to body weight the specific bone mineral density (sBMD) was significantly increased in *C3.Dll1^tm1Gos/+^* animals (Figure 5B). Whole body bone mineral content (BMC) was significantly reduced in *C3.Dll1^tm1Gos/+^* females, and a similar tendency was observed in male animals (Figure 5C). No differences were found when the bone content was related to body weight (Figure 5D). Comparable results were found for heterozygous mutant and wild-type mice of the 129X1 background (data not shown).

### Changes in gene expression patterns

A set of 11 organs from 5 wild-type and 5 heterozygous *C3.Dll1^tm1Gos/+^* male animals was archived for subsequent expression profiling analysis [24]. The brain was selected for the analysis because *Dll1* plays an important role in the differentiation of neuronal precursors [25]. Spleen and thymus were chosen based on the requirement of *Dll1* signaling for immunological parameters [26], [27]. Finally, gene expression in liver was studied due to the importance of the organ in overall energy metabolism. In brain and spleen no significantly regulated genes were detected. 13 significantly downregulated genes were found in the liver of *C3.Dll1^tm1Gos/+^* animals (Table 3). No up-regulated genes were identified in this organ. Among the regulated genes several are associated with metabolic liver functions, like cholesterol biosynthesis.

**Table 3:** Genes regulated in heterozygous *C3.Dll1^tm1Gos/+^* animals when compared to wild-type littermates.

Gene symbol	Entrez ID	Name	Function	Mean log2 ratio
***Genes regulated in the liver***				
*Cdk2*	12566	Cyclin-dependent kinase 2	Regulation of cell cycle	−2.24
*Cth*	107869	Cystathionase	Methionine metabolism	−2.10
*Idi1*	319554	Isopentenyl-diphosphate delta isomerase	Cholestrol biosynthesis	−1.93
*Serpina3k*	20714	Serine peptidase inhibitor, clade A, member 3K	Peptidase inhibitor	−1.89
*Cul2*	71745	Cullin 2	E3 ubiquitin ligase complex	−1.88
*Serpina3c*	16625	Serine peptidase inhibitor, clade A, member 3C	Peptidase inhibitor	−1.87
*Bhmt*	12116	Betaine-homocysteine methyltransferase	Methionine metabolism	−1.87
*Armet*	74840	Arginine-rich, mutated in early stage tumors	Unknown	−1.82
*Serpina3h*	546546	Serine peptidase inhibitor, clade A, member 3H	Peptidase inhibitor	−1.84
*Siah1a*	20437	Seven in absentia 1A	E3 ubiquitin ligase complex	−1.84
*Cyp2f2*	13107	Cytochrome P450, family 2, subfamily f, polypeptide 2	Metabolism of xenobiotics	−1.82
*Hmgcs1*	208715	3-hydroxy-3-methylglutaryl-Coenzyme A synthase 1	Cholesterol biosynthesis	−1.71
*Cyp2c70*	226105	Cytochrome P450, family 2c, polypeptide 70	Metabolism of xenobiotics	−1.70
***Genes regulated in the thymus***				
*mt-Rnr2*		16S rRNA, mitochondrial	Mitochrondrial RNA	−2.68
*2310043N10Rik*	66961		Unknown	−2.20
*Tcrb-V13*	269846	T-cell receptor beta, variable 13	T cell development	−2.11
*Bcl11b*	58208	B-cell leukemia/lymphoma 11B	T cell development	−2.91
*Vldlr*	22359	Very low density lipoprotein receptor	Cholesterol metabolism	−2.90
*Sfrs5*		Splicing factor, arginine/serine-rich 5	RNA metabolic process	−1.69
*Ddx6*	13209	DEAD (Asp-Glu-Ala-Asp) box polypeptide 6	RNA metabolic process	−1.63
*Higd1b*	75689	HIG1 domain family, member 1B	Unknown	−1.67
*AK160221*	AK160221		Unknown	−1.62
*Apc*	11789	Adenomatosis polyposis coli	T-cell development	−1.61
*Trip12*	14897	Thyroid hormone receptor interactor 12	E3 ubiquitin ligase complex	−1.52
*EU433307*			Unknown	−1.50
*Akap8*	56399	A kinase (PRKA) anchor protein 8	Unknown	−1.49
*Ywhab*	54401	Tyrosine 3-monooxygenase protein, beta polypeptide	T-cell development	−1.49

In the thymus of heterozygous mutant animals no up-regulated genes were detected. Instead, 15 genes were significantly downregulated in heterozygous *C3.Dll1^tm1Gos1/+^* animals (Table 3). One third are associated with T cell differentiation or involved in the progress of maturation or migration of T cells and several have unknown functions. Some of the genes regulated in the thymus of heterozygous mutant animals were also found to be regulated in homozygous mutant embryos such as *Ddx6*, *Higd1b* and polypeptides of *Ywha* (14-3-3) complex ([28] and unpublished data).

### Variant phenotype of the missense mutant line C3.Dll1^C413Y/+^

**Figure 6:**
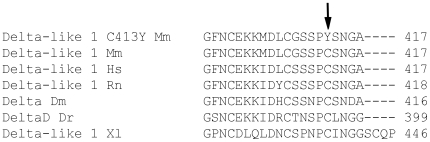
Partial protein sequence alignment of Delta and Delta-like-1 proteins. The sequence alignment was performed with ClutsalW2 [90]. The missense mutation C413Y is indicated by an arrow. The species used for these alignment are: Delta-like 1 Mm, *Delta-like 1 Mus musculus* NP_031891.2; Delta-like 1 Hs, *Delta-like 1 Homo sapiens* NP_114452.1; Delta-like 1 Rn, *Delta-like 1 Rattus norvegicus* NP_005609.3; Delta Dm, *Delta Drosophila melanogaster* NP_477264.1; DeltaD Dr, *DeltaD Danio rerio*; Delta-like 1 Xl, similar to *Delta-like 1 Xenopus laevis*.

*The C3.Dll1^C413Y/+^* mutant line was isolated from an ENU mutagenized combined sperm and genome archive by sequencing the *Dll1* coding region [29]. *C3.Dll1^C413Y/+^* mutants have a missense mutation in exon 8 which leads to the exchange of the cysteine in position 413 by a tyrosine. This mutation lies within the predicted 6^th^ EGF-like repeat, which is highly conserved from insects to vertebrates (Figure 6).

Unlike homozygous *Dll1^tm1Gos^* mutants, homozygous *C3.Dll1^C413Y/C413Y^* mutants are viable, although only 32% of the expected homozygous animals are alive after weaning. *Dll1^C413Y/C413Y^* animals are lighter than their wild-type and heterozygous littermates and are generally in a bad health condition. Most of them died or had to be sacrificed within the first months of life. Since we suspect that the *Dll1^C413Y^* mutation is a partial loss-of-function, we compared the phenotype of *Dll1^C413Y/+^* with *Dll1^tm1Gos/+^* animals (Table 4). Several phenotypes of *Dll1^tm1Gos/+^* mice were also found in *Dll1^C413Y/+^* mice. For example, the body weight and length of heterozygous *C3.Dll1^C413Y/+^* animals was decreased comparable to *C3.Dll1^tm1Gos/+^* mice. In addition, the *C3.Dll1^C413Y/+^* animals show a significantly reduced bone mass density and bone mineral content. The fat mass was also significantly decreased in *C3.Dll1^C413Y/+^* animals. Also comparable to the changes observed in *Dll1^tm1Gos/+^* animals, the systolic, diastolic and mean arterial pressures were significantly increased, and the pulse significantly decreased in *C3.Dll1^C413Y/+^* animals. However, lean mass, locomotor activity, and metabolized energy were increased in *Dll1^C413Y/+^* compared to wild-type littermates. This is somewhat in contrast to *Dll1^tm1Gos/+^* animals, which showed a significant decrease in lean mass and a tendency towards reduced locomotor activity. Concerning the clinical chemical parameters both mutant lines showed similar tendencies in the concentrations of cholesterol and triglycerides in their blood plasma. Whereas *Dll1^tm1Gos/+^* animals showed decreased concentrations of urea, this concentration was significantly increased in the blood of *Dll1^C413Y/+^* animals. Finally, in contrast to the *Dll1^tm1Gos/+^*, no differences were observed in populations of CD19+ and CD4+ cell lines between the *C3.Dll1^C413Y/+^* and wild-type littermates.

**Table 4:** Comparison of the variant phenotypes due to the *C3.Dll1^tm1Gos^* and *C3.Dll1^C413Y^* alleles.

Phenotyping screen	Parameters	Male *C3.Dll1^tm1Gos^* mutant/wt (%)	Male *C3.Dll1^C413Y^* mutant/wt (%)	Female *C3.Dll1^tm1Gos^* mutant/wt (%)	Female *C3.Dll1^C413Y^* mutant/wt (%)
Dysmorpho-logy	Body weight (g)	−19.9***	−9.0**	−12.9***	−14.9***
	Body length (cm)	−4.0*	−2.4*	−3.9***	−4.3***
*DEXA densitometry*	Bone mineral density (mg/cm^2^)	−9.9***	−9.7**	−4.5*	−8.2**
	Bone mineral content (mg)	−14.9^n.s.^	−12.9^n.s.^	−14.0**	−25.3*
	Fat mass (g)	−37.1*	−31.8*	−15.3^n.s.^	−52.2**
	Lean mass (g)	−12.8*	5.8^n.s.^	−12.7***	29.8*
Neurology	Locomotor acitivity (squares)	−14.9^n.s.^	45.8^n.s.^	−10.2^n.s.^	75.0^*^
Metabolism	Food consumption (g/day)	−7.0**	2.2^n.s.^	−22.5**	0^n.s.^
	Metabolized energy (kJ/g×day)	22.8***	12.9***	6.6^n.s.^	17.8***
Cardiovascular	Systolic pressure (mmHg)	10.8**	6.0^n.s^.	5.2^n.s.^	10.9*
	Diastolic pressure (mmHg)	10.8*	7.2^n.s.^	6.0^n.s.^	12.4*
	Mean arterial pressure (mmHg)	10.8*	6.8^n.s.^	5.7^n.s.^	12.0*
	Pulse (bpm)	−2.4^n.s.^	−2.0^n.s.^	−7.6*	−1.2^n.s.^
Clinical chemistry	Urea (mg/dl)	−11.2**	9.1**	−1.5^n.s.^	15.3***
	Cholesterol (mg/dl)	−16.2***	−8.8*	−4.6	−4.7^n.s.^
	Triglyceride (mg/dl)	−23.4**	−35*	−21.1*	−4.4^n.s.^

%: Difference of the means between mutant animals and wild-type in percent. *,**,*** indicates when in the statistical analysis the means were found to be significantly different with a probability of *<0.05. **<0.01 and ***<0.001, ^n.s.^ equals means not statistically significantly different.

## Discussion

The comprehensive investigation of *Dll1* haploinsufficiency reveals a complex phenotype, indicating that several biological processes are affected.

### Immunological phenotype

One of the most studied roles of Notch signaling in adult stages is its involvement in T/B-cell commitment (for review see [26], [27]). Despite a high number of studies, all mechanisms in this process still have not been elucidated. In particular, the role of the Notch ligands is less understood.

Hematopoietic stem cells of the bone marrow give rise to CLP, further proB cells, and immature B cells (in the case that Notch1 is inactive). These cells migrate to the spleen where differentiation occurs. Although we did not detect any differentially regulated gene in the spleen, the frequencies of B- and T-cell populations in the blood of heterozygous animals show a sex-dependent phenotype. As in wild-type mice of different strains under baseline conditions the frequency of T cells is generally higher in females [30], we could interpret our findings as a loss of sex-difference in the size of the T-cell compartment. Whether this might hind to a link of *Dll1* to sex-dependent regulatory mechanisms remains unclear.

Hozumi et al. [31] previously demonstrated that conditional ablation of *Dll1* in hematopoietic cells leads to a disappearance of the marginal zone in the spleen. In the spleen, type 1 transitional B-cells (B1) differentiate into follicular and marginal zone cells. Although not directly investigated, our data affirm the role of Delta-like 1 in B cell commitment and maturation.

The CD8αβ^+^ T cell blood profile of the mutant *Dll1* looks ambiguous. CD8αβ^+^ T cells are significantly increased in male animals but significantly decreased in heterozygous females. Consistent with findings for Notch signaling in lymphocyte development [26], [27] γδ-TCR^+^ cell numbers were not changed in homozygous mutants. Epigenetics has been identified as mechanism in T-cell lineage commitment [32], [33], and could therefore explain the ambiguous variant phenotype observed here.

Nevertheless, *Dll1* haploinsufficiency leads to alterations in the expression of genes, which are clearly linked to T cell differentiation and maturation. For example, *Apc* and *Bcl11b* are downregulated in the thymus. Both genes are essential in maintaining the DN3 checkpoint status, when the cell fate declines either to be αβ- or γδ-T cells [34]–[36]. In the DN3 stage, TCRβ allelic exclusion plays a pivotal role, which is initiated by the pre-TCR complex and controlled by Vβ gene rearrangements. Another gene of the Vβ cluster, *Tcrb-V13*, which is required for efficient Vβ gene rearrangements [37], was also downregulated. Additionally, two further genes, *CD8α* and *Ywhab*, which are associated with T cell differentiation processes [38], [39], were found to be downregulated in *Dll1* haploinsufficient animals.

The blood profile showed reduced CD4^+^ T-cell levels in heterozygous animals, which are significantly decreased only in females. Although a role for Notch signaling in CD8^+^-T versus CD4^+^-T commitment is already described [27], the specific role for Dll1 remains unclear. We found a downregulation of *Trip12* gene expression in thymocytes. *Trip12* is a hect-protein with possibly E3 ubiquitin ligase function [40]–[42]. It is already known, that Notch signaling is highly regulated by ubiquitinylation (for review see [43]). Recently it was shown that double mutants of *Notch* and *Itch*, another E3 ubiquitin ligase from the hect-family, display severely perturbed thymocyte development [44], [45]. The kind of interaction between *Trip12* and *Dll1* has to be elucidated.

In summary, the blood profile and the thymus transcriptomic data affirm an essential role for *Dll1* in lymphoid cell differentiation, maturation and function. *Dll1* haploinsufficiency of the whole organism induces rather a subtle phenotype and the functionality of the *Dll1^C413Y^* allele seems to be sufficient to maintain a normal network function. Probably *Dll1* haploinsufficiency has no immunological pathological consequences, unless the animals are subjected to an immunological challenge. This is in accordance with recent publication, where administration of Delta-Fc (Delta-like 1-immunoglobulin Fc fusion protein) to the lung had an impact on the inhibition of immunological responsiveness, but only in sensitized and challenge animals [46].

### Metabolic phenotype

*Dll1* haploinsufficiency leads to lighter and smaller mice with altered fat and lean mass ratio, higher energy uptake and metabolized energy when normalized to body weight. Hyperactivity seems not to be the reason for the higher energy expenditure. The hyperactive phenotype of *C3.Dll1^C413Y/+^* and *129.Dll1^tm1Gos/+^* females is probably related to a neurological phenotype that should be investigated in detail elsewhere.

Increased blood pressure in haploinsufficient animals may be explained by increased energy demands. Although we did not determine the basal metabolic rate, clinical-chemical parameters in plasma and liver transcriptomic analysis indicate that *Dll1* haploinsufficiency causes alterations of the entire metabolism. The downregulation of genes in the liver is most likely caused by external signals since *Dll1* itself is not expressed in this organ [31], [47]. Thus, the downregulated genes found must be a consequence of signals coming from outside the liver.

Total protein levels in blood plasma were significantly reduced in *Dll1* mutants. For example, the plasma concentration of transferrin, one of the main plasma proteins, was decreased due to *Dll1* haploinsufficiency. Interestingly, nearly all examined immunoglobulins were significantly reduced only in female mutants. The reduction of total protein levels in plasma is subtle and mainly due to the reduction of selected proteins and not caused by a liver disease since the levels of the enzymes alanine-aminotransferase and aspartate-aminotransferase were not altered.

The transcriptional levels of cystathionase (*Cth*) and betaine-homocysteine methlytransferase (*Bhmt*), two enzymes involved in amino acid metabolism, were altered. Cth catalyzes one step in the reactions from homocysteine to cysteine; Bhmt creates methionine from homocysteine. Alterations in the function of both enzymes have been described to alter homocysteine levels [48]–[50]. We hypothesize that a reduction in these transcript levels leads to a reduction of homocysteine levels in plasma rather than to an altered protein metabolism. Consistent with reports on the influence of altered homocysteine levels on bone stability [51], [52] we found a reduced bone mineral density in *Dll1* haploinsufficient animals. Recently it was reported that homocysteine is able to modulate Notch1 function by interacting with cystein bonds in the Notch receptor [50]. Since the Delta-like 1 does not possess cysteine-rich regions the link between the Notch ligand and homocysteine might be indirect.

*Dll1* haploinsufficiency alters also the lipid metabolism. For example, triglycerides and cholesterol are decreased in plasma of *Dll1^tm1Gos/+^* animals. Reduced triglycerides may indicate subtle alterations in lipid handling. *Cyp2c70*, downregulated in the liver of *Dll1* mutants, is involved in linolenic and arachidonic acid metabolism. Of more interest is the downregulation of *Idi1* and *Hmgcs1*, two genes which encode for enzymes involved in cholesterol biosynthesis [53], [54]. Interestingly, low levels of cholesterol leads to high expression of *Adam10*, coding for a protease also known as Kuzbanian, which is essential for Notch activation [55]. Moreover, when *Adam10* is not expressed *Dll1* is overexpressed [56]. Recently, it has been shown that cholesterol is a modulator of γ-secretase activity, and therefore modulates Notch signaling as well [57]. Our results suggest a reciprocal regulation. A decrease in *Dll1* gene expression leads to low cholesterol levels via transcriptional regulation.

Previously, we presented six lines out of our modifier screen with either high cholesterol or transferrin or total protein levels or with altered T- and B-cell patterns, that were all linked to *Dll1* haploinsufficiency [14]. Further studies on these lines will lead to the discovery of several Notch-signaling suppressors.

### Skeletal phenotype

*Dll1* haploinsufficiency leads to a reduction in femoral and whole-body bone mineral density as well as bone mineral content. The role of Notch-signaling pathway components in bone remodeling is known [58]. A close relationship between body weight and bone mass was reported for humans. It was also shown that bone mineral density correlates with metabolic rate and that there is a close connection between lean mass and bone mineral density [59]–[64]. Recent studies in mice demonstrate a reciprocal hormonal link between energy metabolism and bone remodeling [65], [66]. In conclusion, the reduced bone mineral density and bone mineral content in *Dll1* mutants is most probably caused by a reduced body size and an altered metabolic rate.

Cordes *et al.* reported about homeotic transformations with incomplete penetrance in the cervical region of heterozygous *Dll1* mutants [67]. Here we did not observe any alterations in the number of vertebrae linked to *Dll1* haploinsufficiency, due to the small number of animals studied.

### EGF-repeat dependent phenotype

The exchange of an amino acid in the 6^th^ EGF-repeat of the Delta-like 1 protein (C413Y) leads to a phenotype that is, except for alterations in the B- and T-cell patterns, almost identical to the complete loss of an entire *Dll1* allele. The fact that at least a few homozygous *C3.Dll1^C413Y/C413Y^* mutants were born suggests variable doses of functional Delta-like 1 protein.

### Proteolysis mediated Notch signaling regulation

The Notch signaling pathway is mediated by proteolysis and does not seem to be mediated by second messengers (for review see [2]). But some questions remain open. So it is still unclear which E3 ubiquitin ligase monoubiquitinates Notch prior to γ-secretase cleavage [3]. We have already mentioned that *Dll1* haploinsufficiency leads in the thymus to downregulation of a protein that shows E3 ubiquitin ligase function, *Trip12*. However, this is not the only gene found to be downregulated that belongs to the ubiquitin machinery. For example, *Siah1*, which belongs to the E3 ubiquitin ligase complex, is known to promote degradation of Numb, and thereby leads to activation of Notch [68]–[71]. Cul2, another protein of the E3 ubiquitin ligase complex, degrades HIF-1α, whose interaction with NICD leads to transcriptional activation of Notch targets [72]–[74]. As Notch signaling is fine-tuned by different proteases, it can be regulated also by proteases inhibitors. Here we found that due to *Dll1* haploinsufficiency three of the 14 components of the human SERPINA3 (α_1_-antichymotrypsin) homolog cluster were downregulated, *Serpina3c*, *Serpina3h* and *Serpina3k*. Other components of the cluster were not found to be differentially regulated, like *Serpina3a and Serpina3g*. *Serpina3h* and *Serpina3k* probably encode protease inhibitors, whereas *Serpina3c* is more likely an ortholog of kallistatin in humans [75]. Kallistatin is known to have an influence on blood pressure [76], [77].

Taken together, *Dll1* haploinsufficiency leads to a downregulation of selected genes that could function as modulators of the Notch signaling pathway, probably due to feedback mechanisms in maintaining the fine-tuning of this signaling network.

### Link to Wnt signaling

Interactions between Notch and Wnt signaling were first uncovered in the developing wing disc of the fly (for review see [78]). In vertebrates, mutually dependent interactions between Notch and Wnt signaling have been described in several processes like somitogenesis or T-cell development [26], [78]. Wnt signaling activates usually the expression of Notch ligands. The recurrence of this regulatory relationship is higher than the occurrence of regulation in two distinct interacting networks, suggesting that probably Wnt and Notch signaling devices are part of an integrative system called ‘Wntch’ [78]. It is not the aim of this study to investigate this hypothesis. Nevertheless, we detected in this work a relation between the Notch signaling and the Wnt signaling pathway. The two genes, *Apc* and *Siah1*, which were found to be downregulated in *Dll1* mutants, are known to act via β-catenin and by extension on Wnt signaling [34], [35], [70].

### Conclusion

*Dll1* haploinsufficiency leads to a complex phenotype with several biological processes altered. These alterations emphasize the importance of *Dll1* mainly in metabolism, energy balance and in immunology. The *Dll1* haploinsufficient animals are smaller, lighter, with altered fat to lean ratio and have an increased blood pressure and a slight bradycardia. At the immunological level a subtle phenotype is observed due to the effects and fine-tuning of the signaling network at different levels of differentiation, proliferation and function of lymphocytes. Moreover, the importance of the proteolytic regulation of the Notch signaling network is emphasized.

## Materials and Methods

### Mice

Mice were housed and handled according to the federal animal welfare guidelines and all the animal studies were approved by the state ethics committee. Mouse husbandry was conducted under a continuously controlled specific pathogen-free (SPF) hygiene standard according to the Federation of European Laboratory Animal Science Associations (FELASA). For this study we used mice carrying the *Dll1^tm1Gos^* allele on a 129X1/SvJ background [15], and these mice were outcrossed 11 generations to wild-type C3HeB/FeJ mice. The mutant line was further maintained by mating between siblings. The *C3.Dll1^C413Y/+^* mutant line was isolated from an ENU mutagenized combined sperm and genome archive by sequencing the *Dll1* coding region [29]. Phenotypic screening was performed by comparing heterozygous mice with the same number of age- and sex-matched littermate controls [20]. Mice were genotyped for the *Dll1*-*LacZ* insertion as described elsewhere [14]. The isogenicity of the *Dll1^tm1Gos^* in the C3HeB/FeJ background was evaluated with the SNP panel developed at our institute for linkage analysis as described elsewhere [14].

### Metabolic screen

At the age of 17 to 19-weeks wild-type and heterozygous Dll1^tm1Gos^ mutants entered the metabolic screen in the GMC, and were analyzed as described previously [79]–[81]. In the primary screen mice were single-caged and fed ad libitum for a period of 14 days. The following parameters were measured: body weight, food consumption (F_con_), rectal temperature (T_re_), daily faeces production (Fec), energy uptake (E_up_), energy content of the faeces (E_fec_), metabolizable energy (E_met_) and food assimilation coefficient (F_ass_). In the tertiary screen the body composition was analyzed. At a mean of 31 weeks of age the animals were killed, weighed and the gastrointestinal tract was removed. Body composition was determined by drying the dissected carcass, weighing the dry carcasses and fat mass was determined by extracting the lipids using a refluxing Soxhlet apparatus with petrol ether as solvent for 16 hours [82]. The post extraction dry mass was determined. Lean mass was calculated as the carcass weight minus the fat mass.

### Cardiovascular screen

At the age of 14 weeks the mice entered the cardiovascular screen of the GMC [83]. Blood pressure was measured in conscious mice with a non-invasive tail-cuff method using the MC4000 Blood pressure Analysis Systems (Hateras Instruments Inc.). Pulse detection, cuff inflation and pressure evaluation were automated by the system software. At least 20 to 48 individual measurements were pooled to obtain a mean over the four measurements days for each animal. The electrocardiogram analysis (ECG) was performed in isoflurane-anesthetized mice by the use of three metal bracelets on the joints of the feet. Positioning on the front paws and the left hind-paw allows the recording of the bipolar standard limbs I, II, and III and the augmented unipolar leads AVF, AVR and AVL. The ECG was recorded for seven minutes. In the quantitative ECG analysis sets of 5 analyzed beats were averaged for each animal. The shape analysis of the ECG traces was performed with the software ECG-auto (EMKA technologies).

### Clinical chemistry screen

Blood samples were obtained from 12-week-old mice by puncturing the retro-orbital sinus under anesthesia [84]. Plasma was separated by centrifugation (10 min, 4656×g; Biofuge, Heraeus; Hanau, Germany) diluted 1∶2 with deionized water and analyzed in the GMC for clinical chemistry parameters. The 20 parameters measured cover a broad spectrum suitable to investigate alterations in metabolism, organ function and electrolyte homeostasis. In detail the electrolytes sodium, potassium, total calcium, chloride and inorganic phosphorus; total protein in plasma and the proteins ferritin and transferrin; the metabolites creatinine, urea, uric acid, cholesterol, triglycerides and glucose; and the enzymes creatine kinase, alanine-aminotransferase (ALT), aspartate-aminotransferase (AST), alkaline phosphatase, α-Amylase and lipase were analyzed. The parameters were analyzed using an Olympus AU 400 autoanalyzer and adapted reagents from Olympus (Hamburg, Germany), as described elsewhere [85]. The results presented here include individuals investigated under the same analytical conditions.

### Immunology screen

A fraction of the blood sample taken at 12 weeks of age was separated to analyze immunological parameters using ELISA and flow cytometry, as described previously [86]. The following main cell populations were analyzed by flow cytometry: B cells (CD19^+^ clone 1D3), B1 B cells (CD19^+^CD5^+^, clone 53-7.3), B2 B cells (CD19^+^CD5^−^), T cells (CD3^+^, clone 145-2C11), CD4^+^ T cells (clone RM4-5), CD8^+^ T cells (CD8α, clone 53-6.7; CD8β, clone H35-17.2), γ/δT cells (clone GL3), granulocytes (Gr-1^+^, clone RB6-8C5), and NK cells (CD49b^+^, clone DX5). Data were acquired on a LSR II flow cytometer (Becton Dickinson, USA) and were analyzed using FlowJo software (TreeStar Inc, USA). All samples were acquired until a total number of 30,000 cells were reached.

The plasma levels of IgM, IgG_1_, IgG_2a_, IgG_2b_, IgG_3_, and IgA were determined simultaneously in the same sample using a bead-based assay with monoclonal anti-mouse antibodies conjugated to beads of different color regions (Biorad, USA), and acquired on a Bioplex reader (Biorad). The presence of rheumatoid factor and anti-DNA antibodies was evaluated by indirect ELISA with rabbit IgG (Sigma-Aldrich, Steinheim, Germany) and calf thymus DNA (Sigma-Aldrich), respectively, as antigens and AP-conjugated goat anti-mouse secondary antibody (Sigma-Aldrich). Serum samples from MRL/MpJ-Tnfrsf6^lpr^ mice (Jackson Laboratory, Bar Harbor, USA) were used as positive controls in the autoantibody assays.

### Dysmorphology screen

The animals were checked for general condition and health at the age of 5 weeks when entering the GMC. At the age of nine weeks mice were subjected to morphological observation following the protocol described previously [87]. Growth, weight, body size, eye, coat hair growth, coat hair texture, hair follicle structure and orientation, skin pigmentation, skin texture and condition of vibrissae, limbs, digits, tail, teeth, ear morphology, musculature, seizures and epilepsy, motor capabilities and coordination, movement, feeding and drinking behavior, respiratory system, reproductive system or other abnormalities in the body morphology were investigated. X-ray analysis, as well as DEXA scans were performed at the age of 16 weeks. For the X-ray analysis animals were analyzed with the Faxitron X-ray Model MX-20 (Specimen Radiography System, USA). To check bone density alterations the mice were investigated with the pDEXA Sabre X-ray Bone Densitometer (Norland Medical Systems. Inc., UK).

### Molecular phenotyping

At the age of 16 weeks five wild-type and five heterozygous *Dll1^tm1Gos/+^* mutants were killed between 9 and 12 am by carbon dioxide and gene expression in liver, spleen, thymus and brain was analyzed as described previously [24]. Briefly, RNA was extracted using the RNeasy Midi kit (Quiagen) following the manufacturer's instructions. For each cDNA array, 15 µg of total RNA were used for reverse transcription and indirect labeled with the fluorescent dyes. Cy3 or Cy5 (Amersham Biosciences, Freiburg, Germany), according to a modified TIGR protocol [88]. In total our cDNA chips contains 20355 spotted sequences (for a full description see GEO database GPL3697). All experiments were submitted to the GEO database: GSE11867. After hybridization the dried slides were scanned with a GenePix 4000A microarray scanner, and the images were analyzed using the GenePix Pro 6.0 image processing software (Axon Instruments). Statistical analyses were performed using TM4 microarray suite [24], [89].

### Data analysis

For biological interpretation of the data the software tools MetaCore™ (GeneGo Inc., St. Joseph, MI, USA) and BiblioSphere PathwayEdition™ Genomatix Software GmbH, Munich, Germany) were used. The databases Mouse Genome Informatics (MGI, The Jackson Laboratory, USA), MapViewer (NCBI, USA), GeneCards® (Weizmann Institute of Science, Israel), The Kyoto Encyclopedia of Genes and Genomes (KEGG, Kanehisa Laboratories, Japan) and The Comprehensive Enzyme Information System (BRENDA, TU Braunschweig, Germany) were used to further interpret the biological meaning of the data.

### Statistical analyses

All statistical tests were performed using either the software package GraphPad Prism 4.01 (GraphPad Software, USA) or the R Version 2.7.0 for Windows provided by the R Foundation of Statistical Computing. Outliers defined as those falling outside the 25^th^ or 75^th^ quantile and 1.5 times the interquantile range were discarded. The distribution of the data was assessed using the Shapiro-Wilk normality test. The variances were compared using the Levene Test. Samples with continuous data were compared using the two-sample unpaired t-test or the Mann-Whitney test. Samples with categorical data where compared using the Fisher's Exact Test or the Chi-square Test.

## Supporting Information

Table S1 (0.05 MB DOC)

Table S2 (0.04 MB DOC)

Table S3 (0.06 MB DOC)
