# Supplementary material for: Dll1 Haploinsufficiency in Adult Mice Leads to a Complex Phenotype Affecting Metabolic and Immunological Processes
Source: PLoS One. 2009 Jun 29;4(6):e6054. doi: 10.1371/journal.pone.0006054 (PMC2699037; doi:10.1371/journal.pone.0006054)
Supplement: Table S1 — (0.05 MB DOC) [file pone.0006054.s001.doc]

**Table S1.** Body weight and composition parameters in heterozygous *C3.Dll1^tm1Gos/+^* animals and wild-type littermates.

| **Phenotyping screen** | **Sex** | **Parameter** | ***C3.Dll1^+/+^*** | ***C3.Dll1^tm1Gos/+^*** | ***P-value*** |
| --- | --- | --- | --- | --- | --- |
| **Weight in F1 population^a)^** | Male | Weight at 6 weeks old | 22.21 ± 1.78 | 19.53 ± 2.10 | < 0.0001*** |
|  |  | Weight at 8 weeks old | 25.65 ± 2.20 | 22.57 ± 1.86 | < 0.0001*** |
|  |  | Weight at 10 weeks old | 27.77 ± 2.48 | 24.64 ± 2.38 | < 0.0001*** |
|  |  | Weight at 12 weeks old | 29.44 ± 2.67 | 26.28 ± 2.52 | < 0.0001*** |
|  |  | Weight at 15 weeks old | 31.04 ± 3.16 | 26.30 ± 3.04 | < 0.0001*** |
|  | Female | Weight at 6 weeks old | 19.08 ± 1.70 | 16.09 ± 1.71 | < 0.0001*** |
|  |  | Weight at 8 weeks old | 21.80 ± 2.08 | 18.06 ± 1.89 | < 0.0001*** |
|  |  | Weight at 10 weeks old | 23.90 ± 2.49 | 20.60 ± 2.38 | < 0.0001*** |
|  |  | Weight at 12 weeks old | 25.38 ± 2.83 | 21.32 ± 2.16 | < 0.0001*** |
|  |  | Weight at 15 weeks old | 28.13 ± 4.08 | 23.04 ± 3.31 | < 0.0001*** |
| ***DEXA densitometry^b)^*** | Male | Body weight (g) | 33.52 ± 2.58 | 26.86 ± 1.71 | < 0.0001*** |
|  |  | Fat mass (units) | 10.33 ± 4.30 | 6.49 ± 1.44 | 0.021* |
|  |  | Fat content (units×100/g) | 0.30 ± 0.14 | 0.24 ± 0.05 | 0.14 |
|  |  | Lean mass (units) | 19.91 ± 3.01 | 17.36 ± 1.26 | 0.029* |
|  |  | Lean content (units×100/g) | 0.60 ± 0.12 | 0.65 ± 0.05 | 0.26 |
|  | Female | Body weight (g) | 28.96 ± 2.10 | 25.22 ± 1.47 | 0.000297*** |
|  |  | Fat mass (units) | 7.92 ± 1.99 | 6.71 ± 2.13 | 0.21 |
|  |  | Fat content (units×100/g) | 0.27 ± 0.05 | 0.26 ± 0.07 | 0.79 |
|  |  | Lean mass (units) | 17.45 ± 0.79 | 15.24 ± 1.29 | 0.00037*** |
|  |  | Lean content (units×100/g) | 0.61 ± 0.05 | 0.61 ± 0.07 | 0.95 |

Values displayed as mean ± SD. *P-value* calculated performing unpaired t-test when samples normally distributed and with equal variances, if not Mann-Whitney test performed*:* * < 0.05, ** <0.01, *** <0.001.

^a)^ The F1 generation was obtained after crossing C3H ENU mutagenized males (3 × 90 mg /kg body weight) with heterozygous *C3.^Dll1lacZ/+^* females

^b)^ Body composition parameters were measured at 16 weeks of age in the dysmorphology unit at the GMC.
