# Supplementary material for: Dll1 Haploinsufficiency in Adult Mice Leads to a Complex Phenotype Affecting Metabolic and Immunological Processes
Source: PLoS One. 2009 Jun 29;4(6):e6054. doi: 10.1371/journal.pone.0006054 (PMC2699037; doi:10.1371/journal.pone.0006054)
Supplement: Table S2 — (0.04 MB DOC) [file pone.0006054.s002.doc]

**Table S2.** Metabolic parameters from heterozygous *129.Dll1^tm1Gos/+^* animals and wild-type littermates during 2-weeks feeding *ad libitum*.

| **Sex** | **Parameter** | ***129.Dll1^+/+^*** | ***129.Dll1^tm1Gos/+^*** | **P-value** |
| --- | --- | --- | --- | --- |
| Male | Body weight (g) | 30.51 ± 2.08 | 27.90 ± 1.47 | 0.025* |
|  | Food consumption (g/day) | 3.54 ± 0.48 | 3.45 ± 0.56 | 0.75 |
|  | Energy uptake BW^-1^ (kJ/day×g) | 2.14 ± 0.91 | 2.28 ± 0.29 | 0.33 |
|  | Feces (g/day) | 0.76 ± 0.07 | 0.73 ± 0.10 | 0.66 |
|  | Metabolized energy (kJ/day) | 51.91 ± 7.53 | 50.95 ± 8.86 | 0.84 |
|  | Metabolized energy BW^-1^ (kJ/day×g) | 1.70 ± 0.19 | 1.82 ± 0.25 | 0.34 |
|  | Body weight decrease after 2 days fasting (%) | 17.60 ± 1.53 | 21.48 ± 2.02 | 0.0022** |
| Male | Body weight (g) | 23.70 ± 1.33 | 21.74 ± 1.36 | 0.018* |
|  | Food consumption (g/day) | 3.01 ± 0.23 | 3.06 ± 0.24 | 0.74 |
|  | Energy uptake BW^-1^ (kJ/day×g) | 2.34 ± 0.14 | 2.59 ± 0.10 | 0.002** |
|  | Feces (g/day) | 0.58 ± 0.05 | 0.56 ± 0.05 | 0.62 |
|  | Metabolized energy (kJ/day) | 45.39 ± 3.73 | 46.54 ± 3.81 | 0.58 |
|  | Metabolized energy BW^-1^ (kJ/day×g) | 1.92 ± 0.11 | 2.14 ± 0.08 | 0.001** |
|  | Body weight decrease after 2 days fasting (%) | 12.12 ± 2.05 | 13.52 ± 2.16 | 0.24 |

Values displayed as mean ± SD. *P-value* calculated performing unpaired t-test when samples normally distributed and with equal variances, if not Mann-Whitney test performed*:* * < 0.05, ** <0.01, *** <0.001.
