# Supplementary material for: Dll1 Haploinsufficiency in Adult Mice Leads to a Complex Phenotype Affecting Metabolic and Immunological Processes
Source: PLoS One. 2009 Jun 29;4(6):e6054. doi: 10.1371/journal.pone.0006054 (PMC2699037; doi:10.1371/journal.pone.0006054)
Supplement: Table S3 — (0.06 MB DOC) [file pone.0006054.s003.doc]

**Table S3.** Clinical chemical parameters from fed heterozygous *C3.Dll1^tm1Gos^* animals and wild-type littermates.

| Sex | Parameter | ***C3.Dll1^+/+^*** | ***C3.Dll1^tm1Gos/+^*** | ***P-value*** |
| --- | --- | --- | --- | --- |
| Male | Sodium (mmol/l) | 148.57 ± 3.08 | 148.40 ± 3.56 | 0.89 |
|  | Potassium (mmol/l) | 4.67 ± 0.24 | 4.51 ± 0.34 | 0.14 |
|  | Calcium (mmol/l) | 2.39 ± 0.05 | 2.35 ± 0.09 | 0.17 |
|  | Cl (mmol/l) | 107.86 ± 1.99 | 107.35 ± 2.46 | 0.54 |
|  | Phosphorous (mmol/l) | 1.71 ± 0.20 | 1.92 ± 0.27 | 0.029* |
|  | Total Protein (g/dl) | 5.31 ± 0.20 | 5.07 ± 0.25 | 0.00671** |
|  | Creatinine (mg/dl) | 0.35 ± 0.03 | 0.35 ± 0.03 | 0.56 |
|  | Urea (mg/dl) | 54.49 ± 5.06 | 48.48 ± 4.71 | 0.0027** |
|  | Uric acid (mg/dl)^1^ | 1.58 ± 0.25 | 1.29 ± 0.26 | 0.0052** |
|  | Cholesterol (mg/dl) | 151.86 ± 9.98 | 128.19 ± 10.55 | <0.0001*** |
|  | Triglyceride (mg/dl) | 180.51 ± 37.17 | 138.24 ± 39.63 | 0.0064** |
|  | Creatine kinase (U/l)^1^ | 83.14 ± 72.38 | 279.15 ± 428.42 | 0.10 |
|  | ALT (U/l) | 19.57 ± 5.93 | 14.40 ± 5.41 | 0.0208* |
|  | AST (U/l) | 16.86 ± 2.91 | 21.60 ± 12.77 | 0.18 |
|  | Alkaline phosphatase (U/l) | 80.86 ± 4.75 | 84.93 ± 5.85 | 0.05 |
|  | α-Amylase (U/l) | 1956 ± 98.10 | 1750.67 ± 111.62 | <0.0001*** |
|  | Glucose (mg/dl) | 140.67 ± 27.09 | 164.49 ± 20.51 | 0.012* |
|  | Ferritin (ng/dl) | 22.93 ± 6.72 | 22.89 ± 4.96 | 0.99 |
|  | Transferrin (mg/dl) | 138.66 ± 3.97 | 129.11 ± 4.87 | <0.0001*** |
|  | Lipase (U/l) | 52.68 ± 2.61 | 52.07 ± 6.01 | 0.72 |
| Female | Sodium (mmol/l) | 148.80 ± 1.97 | 146.71 ± 2.02 | 0.0089** |
|  | Potassium (mmol/l) | 4.47 ± 0.27 | 4.37 ± 0.22 | 0.31 |
|  | Calcium (mmol/l) | 2.43 ± 0.07 | 2.39 ± 0.05 | 0.09 |
|  | Cl (mmol/l) | 111.25 ± 1.95 | 108.34 ± 1.71 | 0.00022*** |
|  | Phosphorous (mmol/l) | 1.37 ± 0.20 | 1.46 ± 0.25 | 0.33 |
|  | Total Protein (g/dl) | 5.04 ± 0.31 | 4.51 ± 0.20 | <0.0001*** |
|  | Creatinine (mg/dl) | 0.36 ± 0.02 | 0.37 ± 0.02 | 0.88 |
|  | Urea (mg/dl) | 60.84 ± 6.80 | 59.91 ± 7.18 | 0.72 |
|  | Uric acid (mg/dl)^1^ | 1.34 ± 0.25 | 0.99 ± 0.23 | 0.00068*** |
|  | Cholesterol (mg/dl) | 112.21 ± 10.35 | 107.00 ± 9.26 | 0.16 |
|  | Triglyceride (mg/dl) | 234.24 ± 70.38 | 184.77 ± 49.19 | 0.038* |
|  | Creatine kinase (U/l)^1^ | 90.24 ± 78.86 | 247.64 ± 296.13 | 0.074 |
|  | ALT (U/l) | 17.73 ± 6.27 | 16.29 ± 6.07 | 0.53 |
|  | AST (U/l) | 21.60 ± 3.22 | 25.00 ± 13.21 | 0.36 |
|  | Alkaline phosphatase (U/l) | 115.73 ± 11.51 | 120.71 ± 14.39 | 0.31 |
|  | α-Amylase (U/l) | 2042.27 ± 137.40 | 1880 ± 215.69 | 0.021* |
|  | Glucose (mg/dl) | 137.63 ± 17.97 | 137 ± 22.59 | 0.93 |
|  | Ferritin (ng/dl) | 21.20 ± 5.24 | 21.76 ± 6.98 | 0.81 |
|  | Transferrin (mg/dl) | 150.93 ± 3.01 | 142.17 ± 5.72 | <0.0001*** |
|  | Lipase (U/l) | 59.84 ± 5.63 | 62.48 ± 3.54 | 0.14 |

Values displayed as mean ± SD. ^1^To perform the statistical analysis these parameters were transformed by calculating the logarithm to avoid skewness of the data. *P-value* calculated performing unpaired t-test when samples normally distributed and with equal variances, if not Mann-Whitney test performed*:* * < 0.05, ** <0.01, *** <0.001.
